# Postoperative C-reactive protein as a predictive marker for surgical site infection after cesarean section: Retrospective analysis of 748 patients at a Japanese academic institution

**DOI:** 10.1371/journal.pone.0273683

**Published:** 2022-09-09

**Authors:** Kazuko Miyazaki, Seung Chik Jwa, Eri Katayama, Shunsuke Tamaru, Osamu Ishihara, Yoshimasa Kamei

**Affiliations:** Department of Obstetrics and Gynecology, Saitama Medical University, Saitama, Japan; University of Palermo: Universita degli Studi di Palermo, ITALY

## Abstract

Surgical site infection (SSI) is a common but potentially serious maternal complication of cesarean section (CS). C-reactive protein (CRP) can be used in early detection of SSI. However, its predictive value for post-cesarean SSI has never been investigated. This study aims to evaluate the predictive value of CRP for the development of SSI. This was a hospital-based retrospective cohort study of 748 pregnant women who underwent CS at our university hospital between January 2017 and December 2019. CRP was measured on postoperative days 1, 3, and 6. The predictive values of CRP for SSI were evaluated using receiver operating characteristics analysis. Forty-seven (6.3%) patients developed SSI, of whom 38 (80.9%) underwent emergency CS. Serum CRP levels were significantly higher in the SSI group than in the non-SSI group from postoperative day 1 (64 vs. 81 mg/L, p = 0.001); the difference became more evident on postoperative days 3 and 6. The area under the receiver operating characteristic curve (AUC) for CRP on days 1, 3, and 6 was 0.58 (95% confidence interval [CI], 0.49 to 0.68), 0.70 (0.62 to 0.78) and 0.73 (0.65 to 0.81), respectively. The optimal cutoff value for day 3 and 6 CRP was 66.4 mg/L (sensitivity = 76.1% and specificity = 54.4%) and 22.2 mg/L (sensitivity = 76.5% and specificity = 63.2%), respectively. CRP on postoperative days 3 and 6 can be used as a predictive marker for the development of SSI after CS. Further studies to validate the predictive value in different populations is essential.

## Introduction

Cesarean section (CS) is one of the most frequently performed obstetric operations, with the number of operations continuously increasing over the last decade [1]. In Japan, the rate of CS also substantially increased from 10.0% in 1990 to 19.2% in 2010 [1]. CS enables prompt delivery avoiding potential maternal and fetal risks for severe morbidity and mortality. However, CS is also associated with higher risks of potentially fatal maternal morbidity, including postoperative infection, hemorrhage, and thromboembolic disorders, relative to those following vaginal delivery [2]. Postoperative infection is one of these major complications, and CS is reported to have a risk eight times higher than that of vaginal delivery [3].

Surgical site infection (SSI), defined as infection involving the abdominal incision or uterus, is the most major infectious complications after CS. The reported incidence of SSI after CS varies from 3% to 30% [3–7] because of differences in surveillance methods, patient populations, and antibiotic prophylactic practices. Observational studies have reported several risk factors, such as emergency CS [8], premature rupture of membrane (PROM) [9], and maternal obesity [6,8,10,11], and recently, the effectiveness of several preventive methods, such as postoperative antibiotic regimen and operative procedure have also been proposed [5,12]. However, SSI still remains a major complication which leads to extended hospital stays, increased medical assistance with readmission, higher healthcare costs, and increased physical and psychological burden on patients [13].

C-reactive protein (CRP), which is one of the most frequently used inflammatory markers, is produced by hepatocytes and reflects the acute-phase systemic inflammatory response. Secretion starts 4–10 hours after an inflammatory insult and peaks at 48 hours with a short half-life of 19 hours [14]. The marker is reported to be a useful predictive marker for SSI in the fields of abdominal [15,16], orthopedic [16,17], and gynecological surgery [18]. However, no studies to date have investigated the predictive values of CRP for the development of SSI after CS.

Thus, the current study investigated the predictive value of postoperative CRP on day 1, 3, and 6 after CS for the development of SSI in a Japanese academic institution.

## Materials and methods

This is a retrospective cohort study based on the delivery record and medical chart review at our institute. Saitama Medical University Hospital is a tertiary regional perinatal center where deliveries at gestational weeks of 25 or later are managed. In this study, all pregnant women who had CS with a livebirth between January 2017 and December 2019 and were followed up with a postnatal maternal check-up at one month at our institution were considered eligible for the study. This study was approved by the Institutional Review Board of Saitama Medical University Hospital (approval no. 2021–005). Informed consent was waived by the same ethics committee that approved the study (Institutional Review Board of Saitama Medical University Hospital).

At the operation, both abdominal and vaginal sterilization was performed immediately after spinal anesthesia with 10% povidone-iodine disinfectant; 1 g of cefazoline sodium was routinely injected within 30 minutes of skin incision unless the patient was already receiving an antibiotic regimen or had had an allergic reaction to the drug. In PROM cases, antibiotics were given in the following cases: when chorioamnionitis was clinically suspected 24 h after PROM or when cervical dilatation was planned to induce labor. Those patients received an initial dose of 2 g of cefazolin sodium, followed by 1 g every 8 h. Patients who were group B streptococcus (GBS) carriers or unknown cases received an initial dose of 2 g of ampicillin sodium, followed by 1 g every 4 h. After delivery, the abdominal fascia was closed with continuous sutures using 0 monofilament. After incision sites were washed with saline, a dermostitch suture was performed using 4–0 monofilament. Among the analyzed samples, there were no reported cases with subcutaneous drains. After surgery, incision sites were covered with sterile dressings, which were removed on postoperative day 3.

Serum CRP concentration was measured routinely on postoperative days 1, 3, and 6 for all patients receiving CS at the laboratory of the hospital using a calibrated autoanalyzer (Labospect 008; Hitachi, Japan) with Latex agglutination immunoturbidimetry. The lower limit of detection by this instrument was 0.1 mg/L, but considering the possibility of contamination, 1 mg/L was set as the lower limit of detection at our hospital. All blood tests were evaluated in an in-patient setting.

SSI was defined according to the Centers for Disease Control and Prevention (CDC), National Health Care Safety Network (NHSN) criteria [19]. Briefly, SSI was defined as an infection related to a surgical procedure that occurs near the surgical site, within 30 days of the original operation, and classified as incisional SSIs (involving only skin and subcutaneous tissues), deep incisional SSI (involving deeper softer tissues of the incision), and organ/space SSI (including abscess and anastomotic leak for intra-abdominal operations) [19]. In this study population, we only had cases with superficial incisional SSI, which involves only the skin and subcutaneous tissue of the incision within 30 days after the cesarean section. During hospitalization, midwives checked patients’ wounds every day and consulted a physician if SSI was suspected. Incisional SSI was diagnosed by a physician if a wound demonstrated localized warmth, swelling, peri-incisional erythema with pain at the incisional site with or without separation and purulent discharge. Patients were discharged from the hospital following pelvic and wound examination with transvaginal ultrasound by a physician on postoperative day 6 or 7 as a rule, and very few patients were discharged at an earlier stage. At discharge, patients were informed to contact the hospital if there were any abnormal signs from their wound. Patients visited the outpatient department for a 1-month check-up after the delivery at which the wound and pelvic organs were checked again with transvaginal ultrasound by a physician.

For patients induced to undergo labor, a balloon catheter was used for cervical ripening for women with an unfavorable uterine cervix (i.e., Bishop score <6) 1 day before the augmentation. For women with closed cervical os, a penetrating hygroscopic expander made from hydrogel was instead used. After cervical ripening treatment, labor was augmented with the intravenous administration of either oxytocin or prostaglandin F2α.

Patients’ background information, including age, parity, body mass index (BMI) before delivery, group B streptococcus (GBS) colonization, oral steroid medication use, smoking during pregnancy, and maternal medical complications (e.g., diabetes mellitus, asthma, and autoimmune disease) were extracted from the hospital medical charts. Furthermore, delivery information, such as gestational weeks at delivery, PROM, indication for CS, operation information such as operation duration, use of blood transfusion, and postoperative antibiotic use were also reviewed.

Patient characteristics, including delivery information, with respect to the presence of SSI were evaluated using the chi-squared test, Fisher’s exact test, or Student’s t-test as appropriate. Serum CRP levels on postoperative days 1, 3, and 6 in patients with or without SSI were evaluated using Student’s t-test. Since the majority of cases of SSI (38/47 [80.9%]) were observed after emergency CS, the results were evaluated separately from those of elective CS. The predictive accuracy of CRP on postoperative days 1, 3, and 6 for the development of SSI were evaluated by receiver operating characteristics (ROC) analysis, and the cut-off value including the sensitivity and specificity was determined using Youden’s index. Because one SSI case was diagnosed at postoperative day 3, and 13 SSI cases were diagnosed within 6 postoperative days; those SSI cases that occurred either before or on the same day as the blood test were excluded from the analysis. A two-tailed p-value <0.05 was considered statistically significant. All analyses were performed using the STATA MP statistical package version 16.1 (Stata, College Station, TX, USA).

## Results

During the study period, 779 patients underwent CS at our hospital. Among those, the following patients were excluded: five patients because of stillbirth, five patients who underwent hysterectomy, two patients who underwent colorectal surgery at the time of CS due to colorectal perforation or colon cancer, and six patients who were lost to follow-up before the postnatal maternal check-up at one-month. The remaining 761 patients were eligible for analysis. Among those, 13 patients (1.7%) were excluded because of missing postoperative day 1, day 3, or day 6 blood test results. Finally, 748 patients were included in the analysis. Among those, intraoperative complications occurred in five cases: four bladder injuries and one rectal serosal injury. Similarly, postoperative complications other than SSI occurred in three cases: one case each of acute pyelonephritis, pelvic hematoma, and intra-abdominal wall hematoma without infection.

**Fig 1:**
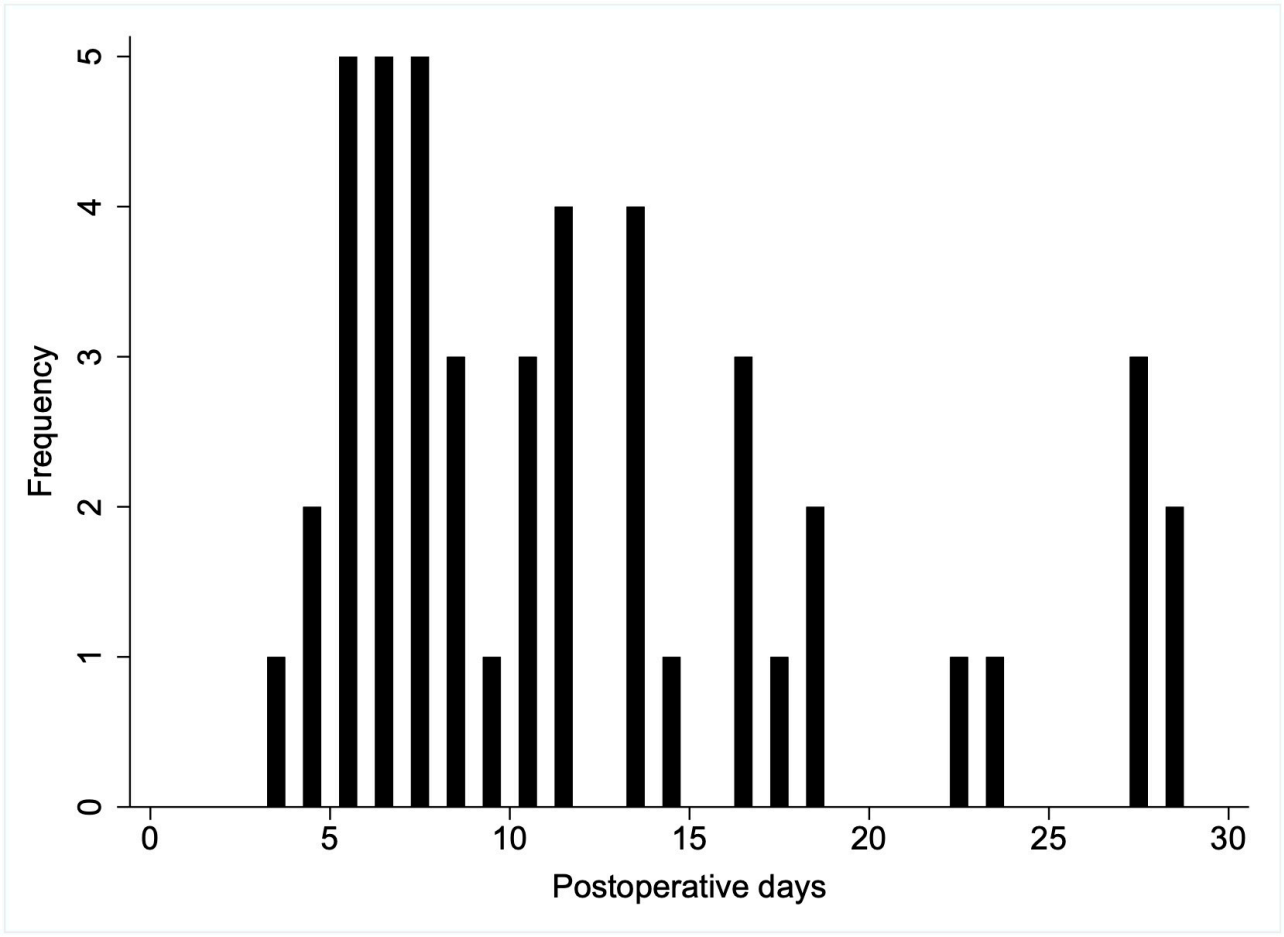
Distribution of number of postoperative days at time of diagnosis of surgical site infection (n = 47).

The distribution of number of days postoperatively until the diagnosis of SSI is shown in Fig 1. Among the 748 patients, 47 (6.3%) were identified as having SSI. The median number of days postoperatively until the diagnosis of SSI was 10 (range 3–29). Thirteen patients (27.7%) developed SSI on postoperative day 6 or earlier.

Patient characteristics stratified by SSI are shown in Table 1. Primiparous patients were significantly more common among patients with SSI (63.8%) than among those without SSI (43.7%) (p = 0.007). Similarly, GBS colonization was more often observed in patients with SSI cases (30.0%) than in those without SSI (15.7%) (p = 0.01).

**Table 1:** Patient characteristics stratified by surgical site infection (n = 748)^a^.

	Non-SSI (n = 701)	SSI (n = 47)	p value^b^
Age (year)	33.5 (5.2)	32.7 (5.4)	0.30
BMI before delivery (kg/m^2^)	26.3 (4.5)	27.3 (5.1)	0.15
Parity			
0	306 (43.7)	30 (63.8)	**0.007**
≥1	395 (56.4)	17 (36.2)	
Maternal Complication			
GDM/DM	92 (13.1)	5 (10.6)	0.24
Asthma	32 (4.6)	5 (10.6)	0.08
Autoimmune disease	14 (2.0)	1 (2.1)	1.00
Psychiatric disease	58 (8.3)	2 (4.3)	0.58
GBS colonization	109 (15.7)	14 (30.0)	**0.01**
Oral steroid intake	14 (2.0)	1 (2.1)	1.00
Smoking during pregnancy	30 (4.3)	3 (6.4)	0.50

^a^ Data are presented as mean (SD) for continuous variables and n (%) for dichotomous variables.

^b^ P-values were assessed using the chi-squared test, Fisher’s exact test, or Student’s t-test.

BMI, body mass index; DM, diabetes mellitus; GBS, group B streptococcus; GDM, gestational diabetes mellitus; SSI, surgical site infection.

Delivery characteristics stratified by SSI is shown in Table 2. Patients with SSI cases more often had PROM (53.2% vs. 18.5%, p < 0.001), induction of labor (27.7% vs. 7.9%, p < 0.001), and chorioamnionitis (27.7% vs. 5.7%, p < 0.001) as indications for CS. Postoperative antibiotics were more frequently used in patients with SSI than in those without SSI (23.4% vs. 11.8%, p = 0.02).

**Table 2:** Delivery characteristics stratified by surgical site infection (n = 748)^a^.

	Non-SSI (n = 701)	SSI (n = 47)	p value^b^
Gestational age at delivery (weeks)	36.7 (2.9)	36.7 (3.5)	1.00
<28	15 (2.1)	2 (4.3)	0.49
28–33	80 (11.4)	7 (14.9)	
34–36	136 (19.4)	9 (19.2)	
≥37	470 (67.1)	29 (61.7)	
PROM^c^	130 (18.5)	25 (53.2)	**<0.001**
<24 hr	85 (65.4)	10 (40.0)	**0.02**
≥24 hr	45 (34.6)	15 (60.0)	
Induction of labor	55 (7.9)	13 (27.7)	**<0.001**
Elective CS	338 (48.2)	9 (19.2)	**<0.001**
Emergency CS	363 (51.8)	38 (80.9)	
Indication for CS^d^			
Previous CS	252 (36.0)	11 (23.4)	0.08
NRFS	70 (10.0)	7 (14.9)	0.28
Breech presentation	94 (13.4)	8 (17.0)	0.49
Placenta previa/low lying placenta	63 (9.0)	1 (2.1)	0.17
HDP	75 (10.7)	8 (17.0)	0.18
Placental abruption	18 (2.6)	1 (2.1)	1
Multiple pregnancy	102 (14.6)	4 (8.5)	0.25
Chorioamnionitis	40 (5.7)	13 (27.7)	**<0.001**
Arrest of labor	36 (5.1)	5 (10.6)	0.17
Other maternal indication	34 (4.9)	1 (2.1)	0.72
Fetal indication	10 (1.4)	0 (0.0)	1
Operational time (min)	61.0 (17.5)	57.4 (15.2)	0.19
Blood transfusion	32 (4.6)	1 (2.1)	0.72
Postoperative antibiotic use	83 (11.8)	11 (23.4)	**0.02**

^a^ Data are presented as mean (SD) for continuous variables and n (%) for dichotomous variables.

^b^ P values were assessed with the use of χ2 or Fisher’s exact test or Student’s t-test.

^c^ Both preterm PROM and PROM were included.

^d^ Multiple answers are allowed.

CS, cesarean section; NRFS, non-reassuring fetal status; HDP, hypertensive disorder during pregnancy; PROM, premature rupture of membrane; SSI, surgical site infection.

CRP on postoperative day 1, day 3, and day 6 stratified by SSI and emergency/elective CS is shown in Table 3. A significantly higher CRP level was observed in patients with SSI on postoperative day 1 (81 mg/L [SD = 50] vs. 64 mg/L [SD = 34], p = 0.001). After stratification by emergency/elective status, elevated CRP in patients with SSI was only observed in those who underwent emergency CS. The significant higher CRP became more evident on postoperative day 3 and day 6. On postoperative day 6, the mean CRP was 48 mg/L (SD = 41) in SSI cases, while that in non-SSI cases was 22 mg/L (SD = 21) (p < 0.0001). A significant increase in CRP in SSI cases was observed following both elective and emergency CS on postoperative day 3 and day 6.

**Table 3:** CRP on postoperative day 1, day 3 and day 6 after cesarean section stratified by surgical site infection and emergency/elective CS^a^.

	Total (n = 748)			Elective CS (n = 347)			Emergency CS (n = 401)		
	Non-SSI (n = 701)	SSI (n = 47)	p value^b^	Non-SSI (n = 338)	SSI (n = 9)	p value^b^	Non-SSI (n = 363)	SSI (n = 38)	p value^b^
CRP (mg/L)									
Day 1	64 (34)	81 (50)	**0.001**	62 (20)	70 (36)	0.22	65 (43)	83 (53)	**0.02**
Day 3	70 (36)	99 (50)	**<0.0001**	67 (31)	104 (58)	**0.0008**	72 (39)	98 (49)	**0.0001**
Day 6	22 (21)	48 (41)	**<0.0001**	21 (17)	36 (20)	**0.009**	24 (25)	51 (45)	**<0.0001**

^a^ Data are presented as mean (SD).

^b^ P-values were assessed using Student’s t-test.

CRP, c-reactive protein; CS, cesarean section; SSI, surgical site infection.

**Fig 2:**
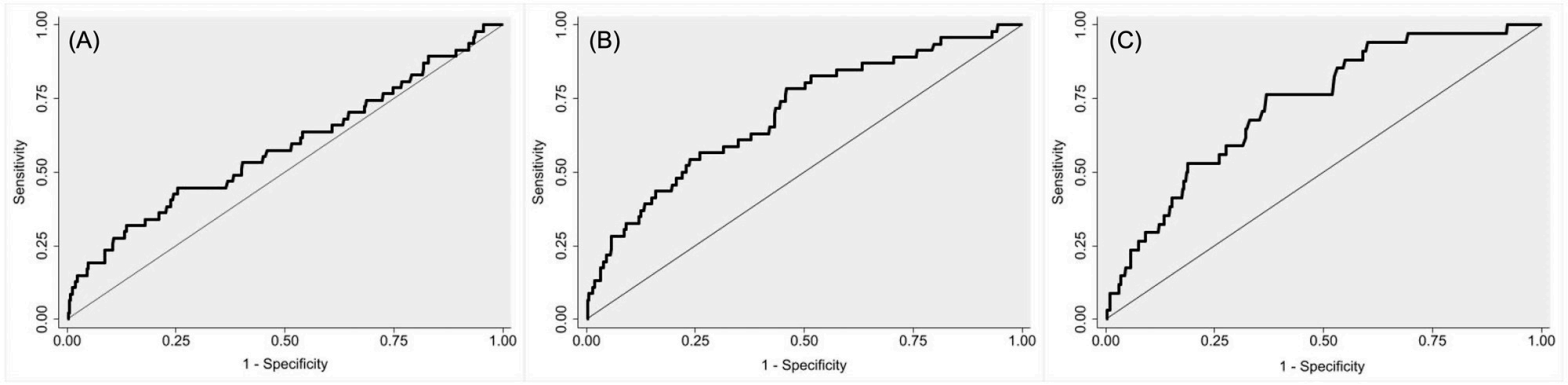
Receiver operating characteristics curves of (A) postoperative day 1 CRP, (B) postoperative day 3 CRP, and (C) postoperative day 6 CRP. Area under the curve for the development of SSI was 0.58 (95% CI, 0.49 to 0.68) for postoperative day 1, 0.70 (95% CI, 0.62 to 0.78) for postoperative day 3, and 0.73 (95% CI, 0.65 to 0.81) for postoperative day 6. The optimal cutoff value for day 3 and day 6 CRP was 66.4 mg/L (sensitivity = 76.1% and specificity = 54.4%) and 22.2 mg/L (sensitivity = 76.5% and specificity = 63.2%), respectively.

ROC curves for postoperative day 1, day 3, and day 6 CRP levels is shown in Fig 2. The AUC of the CRP on day 1, 3, and 6 was 0.58 (95% confidence interval [CI], 0.49 to 0.68), 0.70 (95% CI, 0.62 to 0.78), and 0.73 (95% CI, 0.65 to 0.81), respectively. The optimal cutoff CRP level on days 3 and 6 for SSI was 66.4 mg/L (sensitivity = 76.1% and specificity = 54.4%) and 22.2 mg/L (sensitivity = 76.5% and specificity = 63.2%), respectively.

## Discussion

The current study demonstrates that the magnitude of the postoperative systemic inflammatory response, in particular CRP, is associated with the development of SSI after CS. Furthermore, the threshold of postoperative day 3 and day 6 CRP can predict the likelihood of SSI with moderate diagnostic accuracy at an early clinical stage. CRP on postoperative day 1 could not predict SSI accurately, which may mostly reflect the inflammation caused by surgical trauma. To our best knowledge, this is the first study to evaluate the predictive value of postoperative CRP for the development of SSI.

Our study identified that the majority of cases of SSI (80.9%) occurred following emergency cesarean deliveries. Patients underwent emergency CS for various reasons; however, our study identified patients with PROM, induction of labor, and chorioamnionitis were significantly associated with SSI, indicating pre-existing inflammation before the surgery may affect the incidence of SSI. Previous studies also reported PROM [9], prolonged duration from PROM to delivery [11], and multiple vaginal examinations during labor [8,20] are risk factors for SSI. In the analyzed sample, 45 patients received a balloon catheter and 15 patients received a penetrating hygroscopic expander for cervical ripening due to an unfavorable uterine cervix. Those mechanical procedures used for cervical ripening might also elevate the risk of SSI. Considering that significantly elevated CRP in SSI cases had already been observed on postoperative day 1 among emergency cesarean deliveries (Table 3) and antibiotics were more frequently used postoperatively in SSI cases (Table 2), patients with inflammation before emergency cesarean section may be considered as being at high risk for the development of SSI, regardless of postoperative antibiotic use.

In addition, both postoperative day 3 and day 6 CRP predicted SSI more accurately than day 1 CRP, suggesting that not only pre-operative risk factors and inflammation before surgery, but also postoperative CRP may provide more information regarding the development of SSI after CS. Because CRP reflects the acute phase systemic inflammatory response, a rise in the serum CRP concentration can be considered a sensitive measure of the presence of infection. When considering the clinical application of the postoperative monitoring of CRP levels, those levels will be used in the context of other existing clinical and biochemical parameters. In that sense, monitoring postoperative CRP levels may lead to early investigation of potential infective complications, including SSI, and subsequent preemptive treatment, such as antibacterial medication use or surgical intervention, might be possible. Because of the retrospective, chart-review nature of the current study, no estimation of the value of CRP measurement in promoting earlier clinical intervention for those patients experiencing complications is available. Ideally, a randomized controlled trial investigating the impact of early investigation would resolve this issue.

Although we found no previous studies investigating the predictive value of postoperative CRP for SSI after CS, one study investigated the effect of postoperative inflammatory marker levels on the severity of SSI. Çetin et al. investigated serum procalcitonin (PCT) level, CRP, and white blood cell count among 97 patients who developed incisional SSI after CS [21]. They reported that the serum CRP level was not significantly different between patients who did and did not require secondary sutures. In contrast, serum PCT demonstrated good predictive value for requiring secondary sutures among patients with SSI (AUC = 0.85) and correlated with the length of hospital stay (r = 0.72, p = 0.001). Although it is unrealistic to use PCT as postoperative screening marker because of its high economic cost, serum inflammatory makers can also be used to evaluate the severity of SSI. Postoperative CRP is also reported to be a favorable predictive marker of postoperative complications in patients undergoing laparoscopic shaving for deep infiltrating endometriosis [18].

We evaluated postoperative CRP during hospitalization. However, a wide variety existed for the length of stay after cesarean section around the world [22]. Recent international surveys reported that among 92 countries, the mean length of stay after cesarean delivery ranged from 2.5 to 9.3 days. Especially among western countries, early discharge after CS has become more common to reduce healthcare-related cost. A national register-based study in Denmark reported the length of hospital stay after CS significantly reduced from 97 hours (4.0 days) in 2004 to 58 hours (2.4 days) in 2016 [23]. Furthermore, enhanced recovery after surgery (ERAS) was proposed recently for cesarean deliveries and clinical outcomes and patients’ recognition was comparable to the normal care [24,25]. Thus, further studies to validate the predictive value of postoperative CRP in outpatient clinical settings would be needed before applying the marker where early discharge is common.

Median number of postoperative days until the diagnosis of SSI was 10 in our study, which was comparable to that reported in another study. Wloch et al. reported that among 394 cases of SSI detected in fourteen hospitals in England, the median time to infection following CS was 10 days [6]. Similarly, a prospective study conducted in Brazil using an active telephone monitoring method reported that among 14 cases of SSI, the average time to patients’ notification was 12.2 days [26]. Thus, regardless of the surveillance method, the majority of SSI is detected after discharge and becomes evident within the first 15 days after surgery [10]. Although our study suggested that a 1-month check-up was insufficient for the early detection of postoperative SSI, this check-up is considered important in Japan to evaluate the condition of the mother in the postpartum period, not only to restore the mother’s physical functions, but also for the early detection of postpartum depression and any signs of abuse of the newborn.

The previously reported association between maternal BMI and SSI was not observed in our study. This may be because our study population consisted of fewer obese women than other previous studies. The mean BMI before delivery in our study was 26.4 kg/m^2^, which was much lower than that in other studies [9,11]. Moulton et al. reported a mean BMI in non-SSI cases (n = 2286) of 31.8 kg/m^2^ [11]. Similarly, although several studies reported that maternal GDM/DM is a risk factors for developing SSI [27], there was no such association in our study. This may be because patients diagnosed with GDM/DM are treated intensively during pregnancy in our hospital.

Maternal GBS colonization was significantly more frequently observed in cases of SSI (30.0% vs. 15.7%, p = 0.01), which was inconsistent with a previous U.S. study investigating risk factors for SSI among 2419 pregnant women. Among 113 SSI cases, they reported GBS colonization rates were similar between SSI and non-SSI groups (24.0% vs. 22.9%, p = 0.54) [11]. Since we did not routinely take a bacterial culture from the wound of patients with SSI, the detailed mechanisms and etiology of the relationship between maternal GBS colonization and development of SSI remains unknown. Based on the inconsistent results, further accumulation of cases is necessary.

The strength of our study was that all patients who received CS in our hospital were followed-up according to the hospital’s uniform postoperative protocol. Antiseptic procedures during CS were similar, and CRP on postoperative days 1, 3, and 6 was measured in almost all patients. SSI was monitored intensively during hospitalization and also consistently followed up until the 1-month check-up. These characteristics of the study reduce the bias from the loss to follow-up and missing CRP measurements. However, this study also has several limitations. First, since this was a retrospective study from an academic institution in Japan, our results might not be generalized to other population such as delivery facilities treating low-risk pregnancies or treatment in developing countries. Because our hospital accepted emergency cases, such as placental abruption, from other clinics, several cases of immediate CS without perinatal care at our hospital were included. Similarly, predictive value of postoperative CRP might be different in outpatient settings where patients are discharge from hospital earlier than from our hospital. Third, patients who were suspected of having an infection, such as chorioamnionitis, received antibiotics after the surgery in general obstetric practice, and its effect on postoperative CRPs and predictive value for SSI development is unknown. Thus, the predictive value of postoperative CRP in our study should be interpreted cautiously. Future studies, especially prospective studies, to evaluate the predictive value of CRP and other inflammatory markers after CS in different populations are essential.

In conclusion, this study was the first to evaluate the predictive value of postoperative CRP for the development of SSI after CS. CRP over the threshold on postoperative days 3 and 6, but not postoperative day 1, can predict the likelihood of SSI with moderate diagnostic accuracy. Based on our findings, postoperative CRP can be used as a predictive marker for SSI after CS. Further studies, ideally RCTs, investigating the impact of early diagnosis and preemptive treatment based on the postoperative CRP monitoring are necessary. Our study warrants further prospective studies to validate the predictive performance of post-cesarean CRP levels in a different population.
